# 5,6-Dimethyl-4-(thio­phen-2-yl)-1*H*-pyrazolo­[3,4-*b*]pyridin-3-amine

**DOI:** 10.1107/S1600536812004126

**Published:** 2012-02-04

**Authors:** Hatem A. Abdel-Aziz, Khalid A. Al-Rashood, Hazem A. Ghabbour, Suchada Chantrapromma, Hoong-Kun Fun

**Affiliations:** a Department of Pharmaceutical Chemistry, College of Pharmacy, King Saud University, PO Box 2457, Riyadh 11451, Saudi Arabia; b Crystal Materials Research Unit, Department of Chemistry, Faculty of Science, Prince of Songkla University, Hat-Yai, Songkhla 90112, Thailand; c X-ray Crystallography Unit, School of Physics, Universiti Sains Malaysia, 11800 USM, Penang, Malaysia

## Abstract

In the title mol­ecule, C_12_H_12_N_4_S, the thio­phene ring is disordered over two orientations with a refined site-occupancy ratio of 0.777 (4):0.223 (4). The pyrazolo­pyridine ring system is essentially planar with an r.m.s. deviation of 0.0069 (3) Å and makes dihedral angles of 82.8 (2) and 72.6 (5)°, respectively, with the major and minor components of the thio­phene ring. In the crystal, mol­ecules are linked into a chain along the *a* axis by a pair of N—H⋯N(pyrazole) hydrogen bonds and a pair of N—H⋯N(pyridine) hydrogen bonds, both having a centrosymmetric *R*_2_^2^(8) graph-set motif. A C—H⋯π inter­action is also present.

## Related literature

For bond-length data, see: Allen *et al.* (1987 ▶). For details of hydrogen-bond motifs, see: Bernstein *et al.* (1995 ▶). For background to and bioactivity of pyrazole derivatives, see: Ali (2009 ▶); Bharate *et al.* (2008 ▶); Fu *et al.* (2010 ▶); Thumar & Patel (2011 ▶). For a related structure, see: Fun *et al.* (2011 ▶).
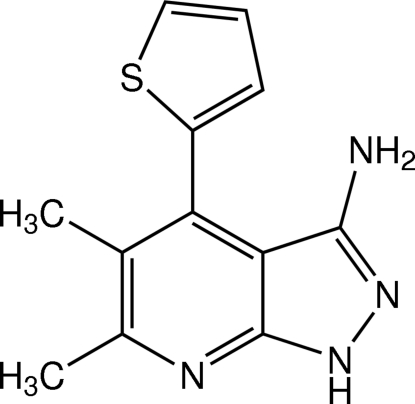


## Experimental

### 

#### Crystal data

C_12_H_12_N_4_S
*M* *_r_* = 244.33
Monoclinic, 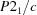

*a* = 10.0688 (2) Å
*b* = 8.0116 (2) Å
*c* = 15.7479 (3) Å
β = 106.809 (1)°
*V* = 1216.06 (5) Å^3^
*Z* = 4
Cu *K*α radiation
μ = 2.22 mm^−1^
*T* = 296 K
0.44 × 0.33 × 0.14 mm

#### Data collection

Bruker SMART APEXII CCD area-detector diffractometer
Absorption correction: multi-scan (*SADABS*; Bruker, 2009 ▶) *T*_min_ = 0.445, *T*_max_ = 0.746
15551 measured reflections
2379 independent reflections
2073 reflections with *I* > 2σ(*I*)
*R*_int_ = 0.040

#### Refinement

*R*[*F* ^2^ > 2σ(*F* ^2^)] = 0.048
*wR*(*F* ^2^) = 0.139
*S* = 1.05
2379 reflections
185 parameters
8 restraints
H atoms treated by a mixture of independent and constrained refinement
Δρ_max_ = 0.46 e Å^−3^
Δρ_min_ = −0.34 e Å^−3^

### 

Data collection: *APEX2* (Bruker, 2009 ▶); cell refinement: *SAINT* (Bruker, 2009 ▶); data reduction: *SAINT*; program(s) used to solve structure: *SHELXTL* (Sheldrick, 2008 ▶); program(s) used to refine structure: *SHELXTL*; molecular graphics: *SHELXTL*; software used to prepare material for publication: *SHELXTL* and *PLATON* (Spek, 2009 ▶).

## Supplementary Material

Crystal structure: contains datablock(s) global, I. DOI: 10.1107/S1600536812004126/is5063sup1.cif

Structure factors: contains datablock(s) I. DOI: 10.1107/S1600536812004126/is5063Isup2.hkl

Supplementary material file. DOI: 10.1107/S1600536812004126/is5063Isup3.cml

Additional supplementary materials:  crystallographic information; 3D view; checkCIF report

## Figures and Tables

**Table 1:** Hydrogen-bond geometry (Å, °) *Cg*1 is the centroid of the C1–C3/N1/C5/C6 ring.

*D*—H⋯*A*	*D*—H	H⋯*A*	*D*⋯*A*	*D*—H⋯*A*
N2—H2*A*⋯N1^i^	0.86	2.08	2.937 (2)	171
N4—H1*N*4⋯N3^ii^	0.93 (2)	2.13 (2)	3.056 (3)	176 (2)
C12—H12*B*⋯*Cg*1^iii^	0.96	2.94	3.717 (2)	139

Symmetry codes: (i) 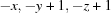; (ii) 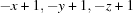; (iii) 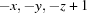.

**Fig. 1.**
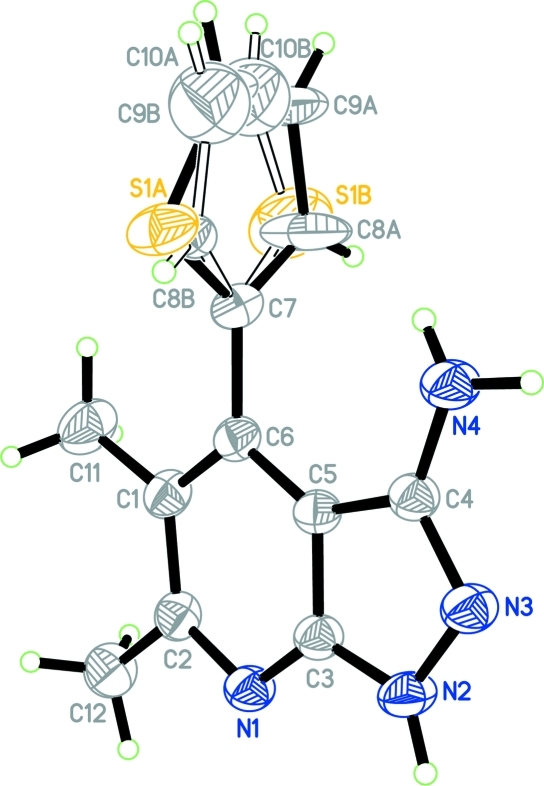
The molecular structure of the title compound, showing 40% probability displacement ellipsoids and the atom-numbering scheme. Open bonds show the minor B component.

**Fig. 2.**
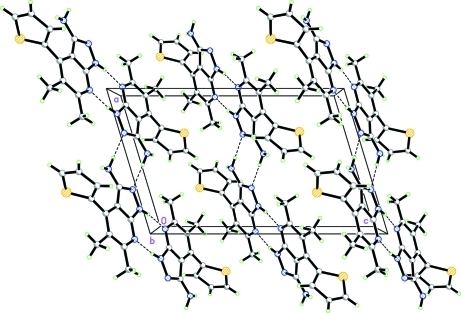
The crystal packing of the title compound viewed along the b axis, showing chains along the [1 0 0]. Only the major component was shown. N—H···N hydrogen bonds are shown as dashed lines.

## supplementary crystallographic information

### Comment

The synthesis of pyrazole derivatives have attracted a lot of interests in medicinal chemistry owing to their biological properties such as anti-cancer (Fu *et al.*, 2010), anti-inflammatory (Bharate *et al.*, 2008) and antimicrobial activities (Ali, 2009; Thumar & Patel, 2011). Pyrazolopyridine, a fused heterocycle, is of interest as a component of potential bioactive molecules. Our on-going research on biological activity of pyrazolone Schiff bases led us to synthesize the title compound (I). Herein, its crystal structure was reported.

In the molecule, C_12_H_12_N_4_S, the thiophene ring is disordered over two positions with the refined site-occupancy ratio of 0.777 (4):0.223 (4). The pyrazolo[3,4-*b*]pyridine moiety (C1–C6/N1–N3) is planar with an *r.m.s*. deviation of 0.0069 (3) Å and the dihedral angle between the pyrazole and pyridine rings is 1.16 (9)°. This planar unit makes dihedral angles of 82.8 (2) and 77.6 (5)° with the major and minor components of the thiophene rings, respectively. The amine and two methyl substituents are co-planar with the pyrazolo[3,4-*b*]pyridine with an *r.m.s*. deviation of 0.0122 (3) Å for the 12 non-H atoms (C1–C6/N1–N4/C11-C12). The bond distances in (I) are within normal ranges (Allen *et al.*, 1987) and comparable to the related structure (Fun *et al.*, 2011).

In the crystal packing, (Fig. 2), the molecules are linked by N2—H2A···N1 and N4—H1N4···N3 hydrogen bonds (Table 1) into cyclic centrosymmetric *R*^2^_2_(8) dimers (Bernstein *et al.*, 1995). These dimers are linked into a chain along the *a* axis (Fig. 2). A weak C—H···π interaction is also observed (Table 1).

### Experimental

A mixture of 2-chloro-5,6-dimethyl-4-(thiophen-2-yl)nicotinonitrile (0.248 g, 1 mmol) and hydrazine hydrate (0.5 mL, 99%) in absolute ethanol (20 ml) was refluxed for 16 h. The reaction mixture was cooled and poured onto ice/water mixture. The precipitate that formed was filtered off, washed with water, dried and crystallized from EtOH/DMF to give yellow crystals of the title compound in 69% yield. Orange block-shaped single crystals of the title compound suitable for X-ray structure determination were recrystallized from ETOH/DMF (3:1 *v*/*v*) by the slow evaporation of the solvent at room temperature after several days.

### Refinement

Amine H atoms were located from the difference map and refined isotropically. The remaining H atoms were placed in calculated positions with N—H = 0.86 Å , and C—H = 0.93 for aromatic and 0.96 Å for CH_3_ groups. The *U*_iso_(H) values were constrained to be 1.5*U*_eq_ of the carrier atom for methyl H atoms and 1.2*U*_eq_ for the remaining H atoms. A rotating group model was used for the methyl groups. The thiophene ring is disordered over two positions with the refined site-occupancy ratio of 0.777 (4):0.223 (4). In the refinement, SAME and FLAT restraints were used for the minor component. The thermal ellipsoids of C9B and C10B were made to be the same.

### Crystal data

**Table:**

C_12_H_12_N_4_S	*F*(000) = 512
*M**_r_* = 244.33	*D*_x_ = 1.334 Mg m^−^^3^
Monoclinic, *P*2_1_/*c*	Cu *K*α radiation, λ = 1.54178 Å
Hall symbol: -P 2ybc	Cell parameters from 2379 reflections
*a* = 10.0688 (2) Å	θ = 4.6–72.1°
*b* = 8.0116 (2) Å	µ = 2.22 mm^−^^1^
*c* = 15.7479 (3) Å	*T* = 296 K
β = 106.809 (1)°	Block, orange
*V* = 1216.06 (5) Å^3^	0.44 × 0.33 × 0.14 mm
*Z* = 4	

### Data collection

**Table:**

Bruker APEX DUO CCD area-detector diffractometer	2379 independent reflections
Radiation source: sealed tube	2073 reflections with *I* > 2σ(*I*)
Graphite monochromator	*R*_int_ = 0.040
φ and ω scans	θ_max_ = 72.1°, θ_min_ = 4.6°
Absorption correction: multi-scan (*SADABS*; Bruker, 2009)	*h* = −11→12
*T*_min_ = 0.445, *T*_max_ = 0.746	*k* = −9→9
15551 measured reflections	*l* = −19→18

### Refinement

**Table:**

Refinement on *F*^2^	Primary atom site location: structure-invariant direct methods
Least-squares matrix: full	Secondary atom site location: difference Fourier map
*R*[*F*^2^ > 2σ(*F*^2^)] = 0.048	Hydrogen site location: inferred from neighbouring sites
*wR*(*F*^2^) = 0.139	H atoms treated by a mixture of independent and constrained refinement
*S* = 1.05	*w* = 1/[σ^2^(*F*_o_^2^) + (0.0775*P*)^2^ + 0.3031*P*] where *P* = (*F*_o_^2^ + 2*F*_c_^2^)/3
2379 reflections	(Δ/σ)_max_ = 0.001
185 parameters	Δρ_max_ = 0.46 e Å^−^^3^
8 restraints	Δρ_min_ = −0.34 e Å^−^^3^

### Special details

**Table:**

Geometry. All esds (except the esd in the dihedral angle between two l.s. planes) are estimated using the full covariance matrix. The cell esds are taken into account individually in the estimation of esds in distances, angles and torsion angles; correlations between esds in cell parameters are only used when they are defined by crystal symmetry. An approximate (isotropic) treatment of cell esds is used for estimating esds involving l.s. planes.
Refinement. Refinement of F^2^ against ALL reflections. The weighted R-factor wR and goodness of fit S are based on F^2^, conventional R-factors R are based on F, with F set to zero for negative F^2^. The threshold expression of F^2^ > 2sigma(F^2^) is used only for calculating R-factors(gt) etc. and is not relevant to the choice of reflections for refinement. R-factors based on F^2^ are statistically about twice as large as those based on F, and R- factors based on ALL data will be even larger.

### Fractional atomic coordinates and isotropic or equivalent isotropic displacement parameters (Å^2^)

**Table:**

	*x*	*y*	*z*	*U*_iso_*/*U*_eq_	Occ. (<1)
N1	−0.02326 (14)	0.27878 (19)	0.44140 (10)	0.0528 (4)	
N2	0.17129 (16)	0.4642 (2)	0.48858 (12)	0.0599 (4)	
H2A	0.1338	0.5370	0.5145	0.072*	
N3	0.30572 (15)	0.4725 (2)	0.48332 (12)	0.0573 (4)	
N4	0.44670 (17)	0.3121 (3)	0.41915 (12)	0.0628 (5)	
C1	0.02348 (19)	0.0354 (2)	0.36199 (12)	0.0529 (4)	
C2	−0.06290 (18)	0.1345 (2)	0.39940 (12)	0.0521 (4)	
C3	0.10716 (17)	0.3269 (2)	0.44769 (11)	0.0479 (4)	
C4	0.32418 (17)	0.3387 (2)	0.43924 (11)	0.0482 (4)	
C5	0.20109 (16)	0.2394 (2)	0.41425 (10)	0.0445 (4)	
C6	0.15813 (17)	0.0883 (2)	0.37008 (10)	0.0464 (4)	
C7	0.2575 (2)	−0.0108 (2)	0.33742 (12)	0.0525 (4)	
C8A	0.3425 (11)	−0.1330 (14)	0.3854 (6)	0.119 (4)	0.777 (4)
H8AA	0.3419	−0.1694	0.4414	0.143*	0.777 (4)
C9A	0.4344 (5)	−0.1978 (6)	0.3355 (3)	0.0991 (16)	0.777 (4)
H9AA	0.4944	−0.2875	0.3540	0.119*	0.777 (4)
C10A	0.4219 (5)	−0.1147 (5)	0.2621 (3)	0.0750 (11)	0.777 (4)
H10A	0.4759	−0.1361	0.2244	0.090*	0.777 (4)
S1A	0.29738 (18)	0.03657 (17)	0.24181 (9)	0.0761 (4)	0.777 (4)
C8B	0.3177 (14)	0.0482 (17)	0.2633 (8)	0.038 (3)*	0.223 (4)
H8BA	0.2986	0.1484	0.2323	0.045*	0.223 (4)
C9B	0.411 (3)	−0.083 (3)	0.2509 (16)	0.131 (9)*	0.223 (4)
H9BA	0.4619	−0.0808	0.2102	0.157*	0.223 (4)
C10B	0.411 (3)	−0.204 (3)	0.3054 (15)	0.131 (9)*	0.223 (4)
H10B	0.4560	−0.3040	0.3027	0.157*	0.223 (4)
S1B	0.3270 (16)	−0.1710 (17)	0.3810 (8)	0.168 (5)	0.223 (4)
C11	−0.0320 (3)	−0.1256 (3)	0.31546 (18)	0.0809 (7)	
H11A	0.0367	−0.1750	0.2919	0.121*	
H11B	−0.1144	−0.1031	0.2680	0.121*	
H11C	−0.0536	−0.2011	0.3569	0.121*	
C12	−0.2079 (2)	0.0787 (3)	0.39368 (16)	0.0687 (6)	
H12A	−0.2504	0.1587	0.4230	0.103*	
H12B	−0.2045	−0.0281	0.4219	0.103*	
H12C	−0.2612	0.0700	0.3325	0.103*	
H1N4	0.524 (2)	0.373 (3)	0.4497 (15)	0.067 (6)*	
H2N4	0.456 (3)	0.214 (4)	0.4039 (19)	0.085 (8)*	

### Atomic displacement parameters (Å^2^)

**Table:**

	*U*^11^	*U*^22^	*U*^33^	*U*^12^	*U*^13^	*U*^23^
N1	0.0438 (7)	0.0573 (8)	0.0612 (8)	−0.0020 (6)	0.0212 (6)	−0.0079 (7)
N2	0.0477 (8)	0.0560 (9)	0.0829 (11)	−0.0053 (6)	0.0296 (8)	−0.0224 (8)
N3	0.0462 (8)	0.0569 (9)	0.0741 (10)	−0.0071 (6)	0.0259 (7)	−0.0156 (7)
N4	0.0468 (8)	0.0693 (11)	0.0791 (11)	−0.0066 (8)	0.0290 (8)	−0.0206 (9)
C1	0.0558 (10)	0.0527 (9)	0.0515 (9)	−0.0068 (7)	0.0177 (7)	−0.0069 (7)
C2	0.0481 (9)	0.0580 (10)	0.0514 (9)	−0.0072 (7)	0.0164 (7)	−0.0038 (7)
C3	0.0445 (8)	0.0484 (8)	0.0533 (9)	−0.0015 (7)	0.0184 (7)	−0.0052 (7)
C4	0.0433 (8)	0.0515 (9)	0.0526 (9)	−0.0011 (7)	0.0187 (7)	−0.0040 (7)
C5	0.0440 (8)	0.0467 (8)	0.0451 (8)	0.0012 (6)	0.0166 (6)	−0.0008 (6)
C6	0.0509 (9)	0.0483 (8)	0.0423 (8)	0.0006 (7)	0.0172 (7)	−0.0014 (6)
C7	0.0592 (10)	0.0499 (9)	0.0530 (9)	−0.0008 (8)	0.0235 (8)	−0.0081 (7)
C8A	0.153 (6)	0.121 (6)	0.117 (5)	0.085 (5)	0.092 (5)	0.027 (4)
C9A	0.118 (3)	0.107 (3)	0.083 (3)	0.069 (3)	0.047 (2)	0.003 (2)
C10A	0.081 (2)	0.073 (2)	0.090 (2)	0.0043 (16)	0.0528 (19)	−0.0218 (18)
S1A	0.0992 (9)	0.0783 (6)	0.0670 (7)	0.0156 (5)	0.0495 (7)	0.0042 (5)
S1B	0.243 (11)	0.120 (5)	0.121 (5)	0.095 (6)	0.023 (5)	−0.004 (4)
C11	0.0786 (15)	0.0764 (14)	0.0926 (16)	−0.0228 (12)	0.0326 (12)	−0.0328 (13)
C12	0.0530 (11)	0.0785 (13)	0.0775 (13)	−0.0143 (10)	0.0235 (10)	−0.0093 (11)

### Geometric parameters (Å, º)

**Table:**

N1—C2	1.334 (2)	C7—S1A	1.709 (2)
N1—C3	1.344 (2)	C8A—C9A	1.472 (6)
N2—C3	1.342 (2)	C8A—H8AA	0.9300
N2—N3	1.381 (2)	C9A—C10A	1.308 (5)
N2—H2A	0.8600	C9A—H9AA	0.9300
N3—C4	1.319 (2)	C10A—S1A	1.706 (4)
N4—C4	1.376 (2)	C10A—H10A	0.9300
N4—H1N4	0.93 (2)	C8B—C9B	1.462 (19)
N4—H2N4	0.83 (3)	C8B—H8BA	0.9300
C1—C6	1.391 (2)	C9B—C10B	1.290 (17)
C1—C2	1.425 (3)	C9B—H9BA	0.9300
C1—C11	1.508 (3)	C10B—S1B	1.669 (17)
C2—C12	1.504 (3)	C10B—H10B	0.9300
C3—C5	1.397 (2)	C11—H11A	0.9600
C4—C5	1.429 (2)	C11—H11B	0.9600
C5—C6	1.401 (2)	C11—H11C	0.9600
C6—C7	1.481 (2)	C12—H12A	0.9600
C7—C8A	1.374 (9)	C12—H12B	0.9600
C7—S1B	1.527 (11)	C12—H12C	0.9600
C7—C8B	1.537 (14)		
			
C2—N1—C3	115.52 (14)	S1B—C7—S1A	112.9 (5)
C3—N2—N3	110.79 (14)	C7—C8A—C9A	110.2 (5)
C3—N2—H2A	124.6	C7—C8A—H8AA	124.9
N3—N2—H2A	124.6	C9A—C8A—H8AA	124.9
C4—N3—N2	106.36 (14)	C10A—C9A—C8A	112.2 (4)
C4—N4—H1N4	118.1 (15)	C10A—C9A—H9AA	123.9
C4—N4—H2N4	113 (2)	C8A—C9A—H9AA	123.9
H1N4—N4—H2N4	120 (2)	C9A—C10A—S1A	113.9 (3)
C6—C1—C2	119.20 (15)	C9A—C10A—H10A	123.1
C6—C1—C11	121.27 (17)	S1A—C10A—H10A	123.1
C2—C1—C11	119.53 (18)	C10A—S1A—C7	91.41 (17)
N1—C2—C1	123.84 (16)	C9B—C8B—C7	106.8 (12)
N1—C2—C12	115.67 (17)	C9B—C8B—H8BA	126.6
C1—C2—C12	120.49 (17)	C7—C8B—H8BA	126.6
N2—C3—N1	126.49 (15)	C10B—C9B—C8B	109 (2)
N2—C3—C5	107.94 (15)	C10B—C9B—H9BA	125.5
N1—C3—C5	125.56 (15)	C8B—C9B—H9BA	125.5
N3—C4—N4	121.28 (16)	C9B—C10B—S1B	117.1 (19)
N3—C4—C5	110.77 (15)	C9B—C10B—H10B	121.5
N4—C4—C5	127.88 (16)	S1B—C10B—H10B	121.5
C3—C5—C6	118.39 (15)	C7—S1B—C10B	94.1 (11)
C3—C5—C4	104.14 (14)	C1—C11—H11A	109.5
C6—C5—C4	137.45 (15)	C1—C11—H11B	109.5
C1—C6—C5	117.49 (15)	H11A—C11—H11B	109.5
C1—C6—C7	122.94 (16)	C1—C11—H11C	109.5
C5—C6—C7	119.52 (15)	H11A—C11—H11C	109.5
C8A—C7—C6	124.5 (3)	H11B—C11—H11C	109.5
C6—C7—S1B	124.2 (5)	C2—C12—H12A	109.5
C8A—C7—C8B	108.5 (6)	C2—C12—H12B	109.5
C6—C7—C8B	123.6 (5)	H12A—C12—H12B	109.5
S1B—C7—C8B	111.7 (7)	C2—C12—H12C	109.5
C8A—C7—S1A	112.1 (3)	H12A—C12—H12C	109.5
C6—C7—S1A	122.78 (14)	H12B—C12—H12C	109.5
			
C3—N2—N3—C4	−0.3 (2)	C5—C6—C7—C8A	90.8 (7)
C3—N1—C2—C1	−0.6 (3)	C1—C6—C7—S1B	−72.2 (8)
C3—N1—C2—C12	178.93 (17)	C5—C6—C7—S1B	105.4 (8)
C6—C1—C2—N1	1.0 (3)	C1—C6—C7—C8B	116.5 (6)
C11—C1—C2—N1	−179.7 (2)	C5—C6—C7—C8B	−66.0 (6)
C6—C1—C2—C12	−178.53 (18)	C1—C6—C7—S1A	103.4 (2)
C11—C1—C2—C12	0.7 (3)	C5—C6—C7—S1A	−79.1 (2)
N3—N2—C3—N1	178.90 (17)	C6—C7—C8A—C9A	−176.1 (5)
N3—N2—C3—C5	0.3 (2)	S1B—C7—C8A—C9A	91 (3)
C2—N1—C3—N2	−178.20 (18)	C8B—C7—C8A—C9A	−16.4 (11)
C2—N1—C3—C5	0.1 (3)	S1A—C7—C8A—C9A	−5.3 (10)
N2—N3—C4—N4	177.36 (18)	C7—C8A—C9A—C10A	5.6 (11)
N2—N3—C4—C5	0.2 (2)	C8A—C9A—C10A—S1A	−3.3 (8)
N2—C3—C5—C6	178.62 (15)	C9A—C10A—S1A—C7	0.2 (4)
N1—C3—C5—C6	0.0 (3)	C8A—C7—S1A—C10A	3.1 (6)
N2—C3—C5—C4	−0.21 (19)	C6—C7—S1A—C10A	174.1 (2)
N1—C3—C5—C4	−178.79 (17)	S1B—C7—S1A—C10A	−9.9 (7)
N3—C4—C5—C3	0.0 (2)	C8B—C7—S1A—C10A	76 (3)
N4—C4—C5—C3	−176.92 (19)	C8A—C7—C8B—C9B	19.9 (13)
N3—C4—C5—C6	−178.47 (19)	C6—C7—C8B—C9B	179.8 (10)
N4—C4—C5—C6	4.6 (3)	S1B—C7—C8B—C9B	7.5 (12)
C2—C1—C6—C5	−0.8 (2)	S1A—C7—C8B—C9B	−91 (3)
C11—C1—C6—C5	179.96 (19)	C7—C8B—C9B—C10B	0.2 (18)
C2—C1—C6—C7	176.76 (17)	C8B—C9B—C10B—S1B	−7 (2)
C11—C1—C6—C7	−2.5 (3)	C8A—C7—S1B—C10B	−87 (3)
C3—C5—C6—C1	0.3 (2)	C6—C7—S1B—C10B	177.9 (10)
C4—C5—C6—C1	178.65 (19)	C8B—C7—S1B—C10B	−9.9 (13)
C3—C5—C6—C7	−177.34 (16)	S1A—C7—S1B—C10B	1.9 (13)
C4—C5—C6—C7	1.0 (3)	C9B—C10B—S1B—C7	11 (2)
C1—C6—C7—C8A	−86.7 (7)		

### Hydrogen-bond geometry (Å, º)

*Cg*1 is the centroid of the C1–C5/N1 ring.

**Table:**

*D*—H···*A*	*D*—H	H···*A*	*D*···*A*	*D*—H···*A*
N2—H2*A*···N1^i^	0.86	2.08	2.937 (2)	171
N4—H1*N*4···N3^ii^	0.93 (2)	2.13 (2)	3.056 (3)	176 (2)
C12—H12*B*···*Cg*1^iii^	0.96	2.94	3.717 (2)	139

Symmetry codes: (i) −*x*, −*y*+1, −*z*+1; (ii) −*x*+1, −*y*+1, −*z*+1; (iii) −*x*, −*y*, −*z*+1.
